# Effects of Modified Biochar on Growth, Yield, and Quality of *Brassica chinensis* L. in Cadmium Contaminated Soils

**DOI:** 10.3390/plants14040524

**Published:** 2025-02-08

**Authors:** Guojun Pan, Shufang Geng, Liangliang Wang, Jincheng Xing, Guangping Fan, Yan Gao, Xin Lu, Zhenhua Zhang

**Affiliations:** 1 Key Laboratory for Saline-Alkali Soil Improvement and Utilization, Ministry of Agriculture and Rural Affairs/Institute of Agricultural Resources and Environment, Jiangsu Academy of Agricultural Sciences, Nanjing 210014, China; pangj202412@163.com (G.P.);; 2 Institute of Jiangsu Coastal Agricultural Sciences, Yancheng 224002, China; 3 School of the Environment and Safety Engineering, Jiangsu University, Zhenjiang 212013, China; 4 The School of Agriculture and Environment, The University of Western Australia, Crawley, WA 6009, Australia

**Keywords:** pakchoi, modified biochar, metal, remediation, passivation, food safety

## Abstract

Cadmium (Cd) pollution in farmland soil leads to excessive Cd in vegetables, which can be transferred to humans through the food chain, posing a significant threat to human health, and requires urgent measures to combat it. Modified biochar may have the potential to remediate Cd pollution in farmland soils. In this experiment, bulk biochar (YC) derived from reed straw or modified biochar by ball milling (Q) either alone or combined with a combination of several passivation agents {potassium hydroxide (K), attapulgite (A), calcium magnesium phosphate fertilizer (M), and polyacrylamide (P)} was applied to soils polluted with Cd, to investigate the growth, yield, and quality of pakchoi (*Brassica chinensis* L.). The results showed that bulk biochar (YC) provided pakchoi with plenty of nitrogen, phosphorus, and potassium, while passivation agents enhance macronutrient accumulation. Compared to YC, modified biochar improved pakchoi yields and nutritional quality. Among them, concentrations of nitrates in pakchoi significantly decreased by 51.8% and 51.0%, while vitamin C levels increased by 29.6% and 19.0%, respectively, in QKAMP and QKAM treatments. The contents of Cd in pakchoi significantly decreased by 21.6% and 18.6%, respectively, in QKAMP and QKAM treatments. The implementation of QKAMP led to the cadmium contents in edible vegetables being lower than the maximum stipulated content as defined by the national standard, but QKAM failed to accomplish it. In conclusion, QKAMP effectively reduced the bioavailability of Cd in the middle to slightly Cd-polluted alkaline soils, making it a suitable soil amendment to improve the yield and quality and mitigate Cd accumulation in vegetables.

## 1. Introduction

Agricultural soil pollution, especially cadmium (Cd), is becoming increasingly severe because of industrialization and population growth [1,2,3]. Cd is highly toxic, reducing soil quality and crop yields, and accumulating in humans through the food chain, posing significant health risks [4]. Given that Cd contaminated agricultural soils are predominantly mild to moderate in China, in situ immobilization techniques are being widely applied to ensure the safety of crops grown on contaminated soils [5,6]. Passivation agents used in in situ remediation are mainly classified into inorganic and organic types. Through adsorption, precipitation, ion exchange, or complex with heavy metals, the bioavailability of Cd in soil can be reduced, which will result in a reduction in the absorption and accumulation of heavy metals by crops [7].

Under limited-oxygen conditions, biochar is produced by pyrolyzing agricultural residues, including straw and manure [8]. As the material has a well-developed porous structure, it is capable of forming clusters that can complex with metal ions, in turn reducing both the bioavailability and mobility of heavy metals in soil [9]. Additionally, biochar contains nutrients that improve soil fertility and boost crop growth [10,11]. However, due to the complexity of soil environments, the sole application of biochar often requires large quantities and incurs high costs. Furthermore, bulk biochar exhibits a limited adsorption ability [12,13]. The adsorption performance of biochar can be significantly enhanced by physically, chemically, or biologically modified biochar [14].

Pakchoi, *Brassica chinensis* L. is a widely cultivated leafy vegetable in China characterized by a short growth cycle, strong environmental adaptability, and high yield. More importantly, pakchoi contains various vitamins and mineral elements essential for human health; thus, it is highly favored by consumers [15]. Heavy metal contamination is more common in leafy vegetables than in other vegetables, according to previous studies [16]. Cultivating pakchoi in cadmium-contaminated soil possibly inhibits its growth while leading to nutrient loss and reduced nutritional quality. Prolonged consumption of pakchoi with elevated cadmium levels poses potential health risks to human beings.

In our previous study, we identified that reed straw biochar modified with either potassium hydroxide or potassium hydroxide combined with attapulgite exhibited favorable effects on the immobilization of Cd in soils [17]. Building upon this foundation, the current study optimizes mechanical ball-milling modifications by incorporating additional functional passivation agents into reed straw biochar. Our study investigated the effects of modified biochar on the growth, nutritional quality, and accumulation of Cd in pakchoi, a model plant for safe vegetable production in soils polluted with Cd.

## 2. Materials and Methods

### 2.1. Preparation of Modified Biochar

Based on reed straw, biochar was prepared for this study [17]. To prepare the straw for use in the future, small sections were cut into smaller pieces, filled with inert nitrogen by heating at the rate of 20 °C per minute and stayed at 600 °C for 30 min, then cooled, ground, and sieved through 60 meshes. Passivating agents used in the experiment included potassium hydroxide (Xilong Chemical Company, Shantou, China), attapulgite (Xuyi Xinyuan Company, Huai’an, China), calcium magnesium phosphate fertilizer (Sanheng Company, Nanning, China), and polyacrylamide (PAM1200-; Xinbang Company, Changzhou, China). The bulk biochar was mixed either alone or combined with the various passivation agents in a specific ratio (see Table 1) and ball-milled at 300 rpm for 4 h. The modified biochar was bagged and stored for use.

### 2.2. Pot Experiment

The tested soil was collected from 0 to 20 cm layers of cadmium-contaminated lands in Shanghu Town, Changshu City, Jiangsu Province, China (120°37′ E, 31°43′ N). A 2 mm screen was used to grind and remove impurities from the soil after it was air-dried and screened. Soil properties are summarized below: pH, 7.07; soil bulk density, 1.32 g·cm^3^; soil organic matter, 24.8 g·kg^−1^; available nitrogen, 41.5 mg·kg^−1^; available phosphorus, 34.8 mg·kg^−1^; available potassium, 74 mg kg^−1^; total Cd, 1.75 mg kg^−1^.

The pot experiment was conducted in the greenhouse of the Jiangsu Academy of Agricultural Sciences (118°52′ E, 32°2′ N). Cylindrical plastic pots (0.15 m in diameter, 0.12 m in height) were used, each filled with 1.5 kg of soil to a depth of 0.10 m. In total, 2% of the modified biochar or bulk biochar (YC) was added to the tested soil (weight percentage) based on our previous studies [17]. Soil without added biochar served as the control (CK). Each treatment was replicated three times. For each pot, 70% of the field water capacity was kept, ensuring uniform mixing while adding water, followed by a two-week stabilization period. After this period, 30 uniformly sized pakchoi seeds, purchased from Mingtian Seed Company in Nanjing, Jiangsu Province, were planted in each pot. After germination, 10 evenly spaced seedlings were retained for further growth. During the cultivation period, watering was performed quantitatively to ensure adequate moisture content (70%) in the soil. After sixty days of growth, plants were harvested to measure both aerial and root fresh weights. Subsequently, representative fresh samples were taken for quality assessment before being dried and ground through a 100-mesh sieve for analysis.

### 2.3. Measurement Methods

#### 2.3.1. Soil Analysis

A pH meter (FE28, Mettler Toledo, Changzhou, China) was used to measure the pH values of these solutions [18]. Based on Kjeldahl’s method, we determined the soil’s nitrogen content. According to Qian et al. [19], volumetric potassium dichromate analysis was used to assess organic matter content; using molybdenum–antimony colorimetry, we measured the phosphorus availability, while flame photometry was used to determine the potassium availability. The soil available Cd concentration (DTPA−Cd) was determined following national standards of China (GB/T 23739-2009) [20].

#### 2.3.2. Plant Parameters

An instrument was used to measure plant height, starting at the soil surface and extending to the highest point of the leaves of the pakchoi plant. Pakchoi was harvested, cleaned with deionized water, air-dried, and divided into aerial parts and roots. Fresh weight was measured using a precision balance. Following 30 min of enzyme deactivation at 105 °C, samples were dried at 70 °C to a constant weight. Measured were the dry masses of aerial parts and roots.

#### 2.3.3. Nutrition Contents in Plants

Plant samples underwent digestion using H_2_SO_4_-H_2_O_2_ to prepare them for analysis. According to Pandey et al. [21], Kjeldahl’s method semi-micro was used to quantify total nitrogen content; molybdenum–antimony colorimetry and flame photometry were used to measure the total phosphorus and potassium levels, respectively.

#### 2.3.4. Plant Quality

The chlorophyll content was measured using spectrophotometric methods [22]. The soluble protein level was evaluated employing Coomassie Brilliant Blue G-250 staining [23]. Nitrate concentration was determined utilizing salicylic acid methods [24]. Soluble sugar content was analyzed through anthrone colorimetric techniques [25]. Vitamin C level was quantified via titration with 2,6-dichlorophenol indophenol [26].

#### 2.3.5. Cadmium Content in Plants

The determination of the total Cd concentration in plants followed the national standards of China (GB 5009.15-2023) [27], which involved sample digestion utilizing a pressure digestion vessel. In a pressure vessel, 0.5 g of dried plant matter was sealed, soaked, and sealed in nitric acid overnight. Cd content was measured via inductively coupled plasma mass spectrometry (PerkinElmer Syngistix, Waltham, MA, USA) after the sample was digested at a constant temperature.

### 2.4. Data Statistical Analysis

Data processing was performed using Microsoft Excel 2019. One-way analysis of variance (ANOVA) was conducted via SPSS 20. Duncan method was used to statistically analyze the significant differences between treatments (*p* < 0.05). The graphs were created using Origin 2021.

## 3. Results and Discussions

### 3.1. Physicochemical Properties and Cd Bioavailability in Soils

Using modified biochar as a soil amendment, Table 2 summarizes its results on physicochemical properties. Soil pH, a fundamental indicator of soil chemistry, greatly influences the form and availability of heavy metals [28]. This study used soil that was mildly alkaline. Compared to CK, the soil pH significantly (*p* < 0.05) increased by 0.20−0.71 units after addition of the biochar. The pH values for soils treated with QKAMP, QKAM, QKM, and QK increased by 0.71, 0.65, 0.63, and 0.69 units, respectively. Furthermore, the pH values were significantly (*p* < 0.05) higher in the biochar treatments including ball milling with the agents than in both Q and YC treatments. The alkali ions of biochar, such as Ca^2+^, Mg^2+^, and K^+^, are released into the soil in the form of carbonates and oxides, which are exchanged with acidic ions to increase the soil pH [29]. Furthermore, the carboxyl, hydroxyl, and phenolic groups on the surface of the biochar can interact with acidic ions, thus helping to reduce the concentration of H^+^ [30]. The passivation agents used in the experiment contain a rich variety of basic functional groups, resulting in a significantly higher pH value in the soil treated with the passivation agents compared to Q and YC.

Compared to CK, total nitrogen contents significantly (*p* < 0.05) increased by 5.3−8.5% among the biochar treatments. In biochar-treated soils, nitrogen was provided, though the effect was not considerable, and the higher nitrogen content may be due to a reduction in nitrogen loss [31]. Soil organic matter contents were also significantly increased by 126.3−146.1% in the biochar treatments, though the modification of biochar did not further enhance organic matter levels. The results of our study are in agreement with those of Mohamed et al. [32], which showed that the organic matter contents in the treatments with 1.5% biochar added to the soil were more than twice that of CK. Moreover, soil available potassium contents were significantly increased by 22.9−489.8% among the different treatments, with addition of KOH being a key factor in enhancing potassium availability. The soil available phosphorus content was 34.86 mg/kg in CK treatment; while bulk biochar (YC) and ball-milled biochar (Q) did not significantly improve the available phosphorus levels. However, the available phosphorus increased by 66.9%, 49.9%, 22.3%, and 20.3%, respectively, in soils treated with QM, QKM, QKAM, and QKAMP, due to the phosphorus in the modifying agents. Previous studies have found that calcium magnesium phosphate, potassium hydroxide, and other modifiers work together with the ball-milled biochar to slow down the loss of nutrients, increased the utilization rate, and ensured the effective growth of subsequent plants [33,34,35]. In addition, bulk biochar reduced available Cd by 10.4%; however, the modified biochar reduced it by 14.4% to 25.5%, when compared with CK. Qin et al. [36], who found that pH and organic matter affect heavy metal bioavailability, a finding that is consistent with our results.

### 3.2. Growth of Pakchoi

#### 3.2.1. Effects of Modified Biochar on Chlorophyll Contents in Pakchoi

**Figure 1:**
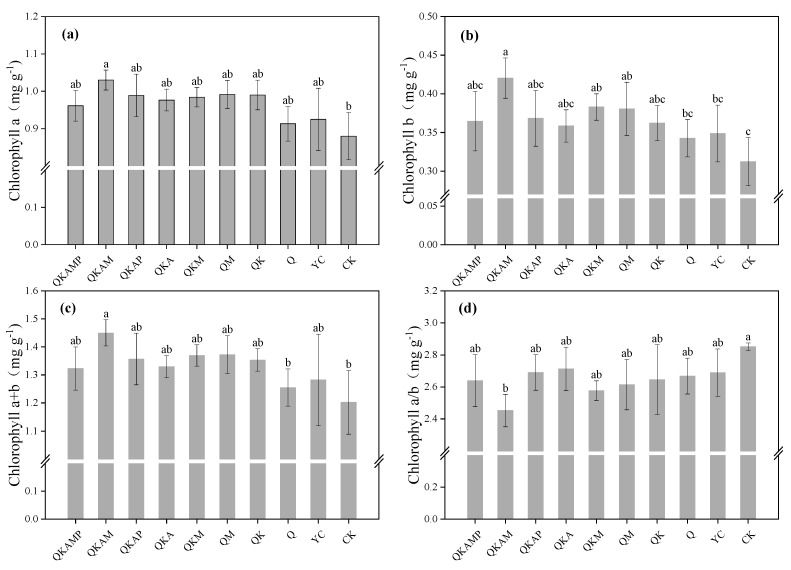
Effects of modified biochar on chlorophyll a (**a**), chlorophyll b (**b**), chlorophyll a + b (**c**), and chlorophyll a/b (**d**) contents in pakchoi. Note: Error bars represent the standard deviation of triplicate samples. Different letters above bars indicate a significant difference at *p* < 0.05.

**Figure 2:**
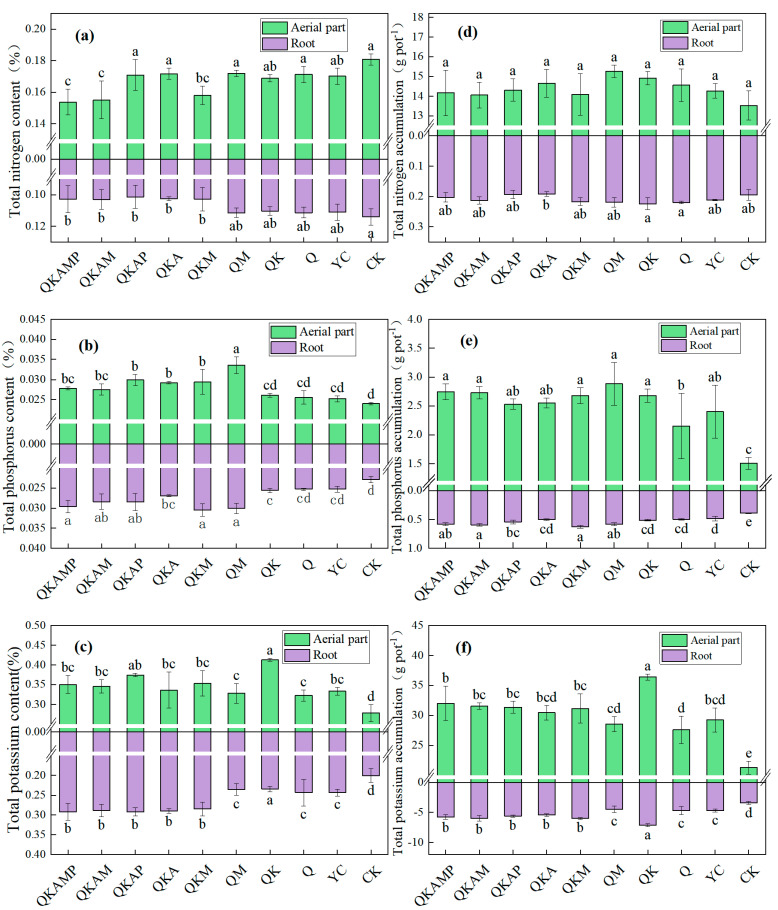
Effects of modified biochar on the content of total nitrogen (**a**), total phosphorus (**b**) and total potassium (**c**) in different parts of pakchoi and the total accumulation of nitrogen (**d**), total phosphorus (**e**), and total potassium (**f**) in different parts of pakchoi. Note: Error bars represent the standard deviation of triplicate samples. Different letters above bars indicate a significant difference at *p* < 0.05.

Chlorophyll directly participates in photosynthesis and thus influences plant growth and yield [37]. The chlorophyll a and b content of pakchoi in this experiment was shown in Figure 1a,b. Modified biochar increased chlorophyll a and b contents to varying degrees. Compared to CK, only chlorophyll a contents in QKAM significantly increased by 14.6%, while bulk biochar, ball milling alone, or with various passivation agent treatments showed insignificant effects. By comparison, chlorophyll b contents in QM, QKM, and QKAM significantly increased by 21.9%, 22.6%, and 34.6%, respectively, compared to CK. Total chlorophyll contents (chlorophyl a + b) increased by 4.3−20.6% compared to CK, and the addition of modifying agents showed a tendency to enhance the photosynthetic capacity of pakchoi, with only QKAM producing a significant effect. (Figure 2c). The chlorophyll a/b ratio, as shown in Figure 2d, was significantly different between QKAM and CK treatments. Biochar has been demonstrated to increase chlorophyll content in plants in previous studies [38,39]. The modifying agents further enhanced the photosynthetic capacity of pakchoi, with QKAM showing the greatest improvement in chlorophyll contents. Cadmium stress inhibits photosynthesis in plants, and the reduction of cadmium accumulation in plants may be an important factor in the elevated chlorophyll content of pakchoi. Several studies have reported that insufficient phosphorus in plants affects the photosynthesis process and thus reduces the chlorophyll contents in plants [40,41]. Battie-Laclau et al. [42] reported adequate potassium enhances carbon dioxide transport within leaves, promoting photosynthesis. Generally, adding modified biochar to soil improved the nutrient content and promoted photosynthesis.

#### 3.2.2. The Effects of Modified Biochar on Plant Height, Root Length and Biomass of Pakchoi

Table 3 shows the response of pakchoi to modified biochar in terms of growth and biomass. The application of biochar significantly improved plant growth compared to the application of CK, which was in line with previous studies that had been conducted [43,44]. The plant height significantly increased in QKAMP, QKAP, QKM, QM, Q, and YC treatments, compared to CK. The root length significantly increased by 1.03−2.07 cm after application of the modified biochar, compared to YC, representing a growth increase of 12.1−24.4%. Compared with the YC treatment, the ball-milled biochar treatment had significantly (*p* < 0.05) longer roots than the YC treatment. Due to its porous structure and high surface area, biochar is able to absorb nutrients and retain water, thus promoting the development of roots [45,46]. The fresh and dry weight of plant roots ranged from 12.54−13.78 g and 1.87−2.07 g, respectively, representing increases of 6.6−17.2% and 9.3−21.1% compared to CK. The fresh and dry weights of arial parts also increased by 10.2−20.7% and 12.9−22.5%, respectively, with significant biomass improvements across the biochar treatments. The biochar in nature contains a variety of nutrients, such as nitrogen, phosphorus, and potassium, all of which improve plant growth and are useful for use as a soil fertilizer when applied to the soil. No additional fertilizers were added in this study aside from the modified biochar materials. Rasul et al. [47] concluded that biochar modulates soil microbial communities to improve plant resistance to stress, thereby increasing crop yields. In addition, the raw materials used in biochar preparation, as well as its application, can significantly affect plant growth.

### 3.3. Contents of Macronutrients in Pakchoi

Figure 2 illustrates the variations in nitrogen, phosphorus, and potassium contents in aerial parts and roots. The total nitrogen contents decreased in the aerial parts treated with biochar (Figure 2a), with significant differences being observed between QKAMP, QKAM, QKA, and CK (*p* < 0.05). Nitrogen is a critical factor in protein and nucleic acid synthesis in plants, and nitrogen deficiency can inhibit normal plant growth [48]. In this experiment, all biochar-treated plants showed a varying degree of reduced nitrogen contents, compared to CK. Biochar contains high levels of carbon, which might immobilize nitrogen and reduce nitrogen loss from the soils [49,50]. However, a high C/N ratio lead to competition between soil microbes and plant roots for available nitrogen, potentially reducing nitrogen uptake by plants [51]. Additionally, biochar has fertilizer retention properties that reduce the rate of nitrogen released from the soil, which could result in inadequate nitrogen supply for short-cycle vegetables like pakchoi. Total phosphorus contents in the aerial parts of biochar-treated plants increased by 5–40%, compared to CK (Figure 2b), with significant (*p* < 0.05) differences being observed in treatments containing calcium magnesium phosphate fertilizer (M). As seen in Table 3, the yield of QKM was also the highest among all the treatments, indicating that calcium magnesium phosphorus fertilizers have a positive effect in promoting higher yields of pakchoi. The potassium contents in pakchoi significantly increased among the biochar treatments, compared to CK (Figure 2c), with QK-treated plants showing a 1.5-fold increase. The nutrient supply capacity of biochar itself is relatively limited, which can be remedied by the addition of modified materials. Nutrient contents in the roots followed a similar trend, with total nitrogen decreasing by 2.3–11.1%, and total phosphorus and potassium increasing by 10.5–33.2% and 17.0–77.6%, respectively. Compared to CK, YC increased total phosphorus and potassium by 10.5% and 21.5%, respectively. Biochar not only supplied nutrients for plant growth but also reduced nutrient losses from modifying agents, improving nutrient uptake efficiency [52,53].

The total nitrogen accumulation in both the aerial parts and roots parts in biochar application treatments did not differ significantly from CK, indicating that bulk/modified biochar did not increase total nitrogen accumulation in pakchoi (Figure 2d). In contrast, total phosphorus and potassium accumulation increased significantly compared to CK (Figure 2e,f). Total phosphorus accumulation in the aerial parts of biochar-treated plants ranged from 2.14 to 2.88 g pot^−1^, representing increases of 42.7−92.0%, compared to CK. The total potassium accumulation in the aboveground portion of pakchoi was also augmented by 29.7–79.0% as a consequence of the incorporation of modified biochar in comparison with CK. Total phosphorus and total potassium accumulation in the roots increased by 23.1–61.5% and 31.1–108.8%, respectively, compared with CK. The addition of modifying agents enhanced phosphorus and potassium contents in the plants, leading to increased nutrient accumulation. Based on the above results, we found that the nutrient supply capacity of biochar itself was relatively limited, and the addition of modified materials could make up for this defect. Compared with YC, ball-milling modification of biochar further improved nutrient utilization and nutrient accumulation in pakchoi.

### 3.4. Nutritional Quality of Pakchoi

#### 3.4.1. Effects of Modified Biochar on Soluble Protein Contents in Pakchoi

**Figure 3:**
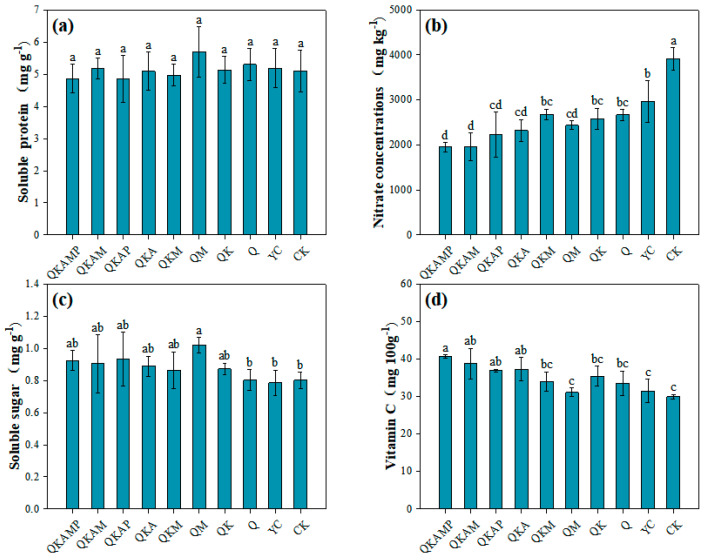
Effects of modified biochar on soluble protein content (**a**), nitrate content (**b**), soluble sugar content (**c**), and vitamin C content (**d**) in pakchoi. Note: Error bars represent the standard deviation of triplicate samples. Different letters above bars indicate a significant difference at *p* < 0.05.

Soluble proteins play a vital role in osmotic regulation and nutrient supply in plants. Their exceptional water-holding capacity not only helps maintain the integrity of plant cell membranes but also promotes plant growth. Increasing soluble protein content could enhance plant stress resistance [54]. The effects of the different modified biochar treatments on soluble protein contents in pakchoi were shown in Figure 3a. Bulk/modified biochar and CK did not exhibit any significant differences in soluble protein content, implying that the supplementation of biochar or biochar incorporating with inactivation agent did not conspicuously enhance the soluble protein content of pakchoi. Nitrogen levels in the environment directly influence soluble protein content [55]. As determined by this experiment, biochar-treated soils contained significantly more nitrogen than control soils, even though the amount of nitrogen accumulated in aerial parts and roots was not statistically significant. This indicated that it was not readily absorbed and utilized by the plants, as biochar increased nitrogen contents in the soil. According to Bi et al. [56], biochar and organic fertilizers can reduce soil N loss and increase vegetable soluble protein levels by a significant amount.

#### 3.4.2. Effects of Modified Biochar on Nitrate Content in Pakchoi

Human health may be compromised by prolonged consumption of foods with high nitrate levels [57]. Moreover, 80% of the nitrates ingested by humans are obtained from vegetables; thus, reducing the nitrate levels in vegetables is of paramount importance [58]. According to Figure 3b, modified biochar affects the nitrate content of pakchoi. Pakchoi’s nitrate content was significantly reduced by the addition of modified biochar, and the order of magnitude of nitrate contents in aerial parts of plants among the treatments was QKAMP < QKAM < QKAP < QKA < QM < QK < Q < QKM < YC < CK. Compared to CK, nitrate contents decreased by 46.7% and 32.1%, respectively, in YC and Q treatments, indicating that ball-milled biochar more effectively reduced nitrate uptake in pakchoi. It was found that the treatments differed significantly, with QKAMP, QKAM, QKAP, and QKA treatments having significantly low nitrate contents. The unique layered structure of attapulgite enhanced biochar adsorption capacity, allowing nutrients like nitrogen to be slowly released into the soil [59]. The nitrate contents in treatments of QKAMP and QKAM were recorded at 1954 mg kg^−1^ and 1964 mg kg^−1^, respectively, with reductions of 100.4% and 99.3%, compared to CK (3916 mg kg^−1^), demonstrating that these treatments substantially decreased nitrate accumulation within pakchoi. Previous studies have shown that nitrate contents in crops was closely associated with nitrogen fertilizer application, with an excessive application of nitrogen fertilizer resulting in a sharp increase in nitrate contents in leafy vegetables [60,61,62]. In this experiment, no additional nitrogen fertilizer was applied, and the modified materials used had low nitrogen content, which explained why no significant increase in nitrate contents was observed. Furthermore, the rich functional groups and porous structure in QKAMP and QKAM treatments provided more binding sites for ammonium and nitrate nitrogen [63], ensuring a stable nitrogen absorption rate by the plants, thereby reducing nitrate accumulation. Yao et al. [64] demonstrated that increasing the pyrolysis temperature of biochar to 600 °C enhances nitrate adsorption, reducing nitrate uptake by plants, which is agreeable with our results. Additionally, as a consequence of Cd stress, nitrate reductase, an enzyme that is crucial in nitrogen assimilation, may experience reducing activity under Cd stress, affecting nitrate reduction and assimilation [65,66]. Both Marousek et al. [67] and Cao et al. [68] concluded that adding biochar to the soil could increase nitrate reductase activity in plant roots, lowering nitrate concentration in plants.

#### 3.4.3. Effects of Modified Biochar on Soluble Sugar Contents in Pakchoi

Soluble sugars are essential sources of energy and carbon for plants, playing a role in energy transfer and regulation during plant growth [69]. Based on Figure 3c, there were no significant (*p* > 0.05) differences in soluble sugar contents between CK and YC or Q, indicating that bulk biochar (YC) and ball milling alone could not enhance soluble sugar contents in pakchoi. By comparison, the treatments with modified biochar increased soluble sugar contents to varying degrees, with QM treatment showing a significant (*p* < 0.05) increase by 29.9% and 28.6% in contrast to YC and CK. The different modifications of biochar, however, did not show any significant differences. Zhang et al. [70] reported that applying calcium magnesium phosphate fertilizer increased soluble sugar contents in bananas. Wang et al. [71] applied calcium fertilizer to two different varieties of apples, finding that soluble sugar content in the harvested fruit increased by 6.3% and 12.0% compared to the control. The application of calcium magnesium phosphate fertilizer enhances nutrient contents in plants and promotes the transport of photosynthetic products and the accumulation of carbohydrates, resulting in the increase in soluble sugar contents [72]. In this study, the modifier—magnesium phosphate fertilizer—played a key role in increasing soluble sugar contents, with QM performing best because of the addition of the highest amount of calcium magnesium phosphate. Bulk biochar also contributed by providing numerous adsorption sites, reducing the loss of mineral elements, and improving nutrient uptake efficiency in pakchoi.

#### 3.4.4. Effects of Modified Biochar on Vitamin C Content in Pakchoi

A natural antioxidant, vitamin C, is present in many plant-based products and is important for human health [73]. Figure 3d illustrates how modified biochar affects the vitamin C content of pakchoi. Compared to CK, the addition of modified biochar increased vitamin C contents in pakchoi. Specifically, the vitamin C contents increased by 36.0%, 24.9%, 22.1%, and 22.6%, respectively, in QKAMP, QKAM, QKAP, and QKA treatments compared to CK. The vitamin C contents increased by 29.6%, 19.0%, 16.3%, and 16.8%, respectively, in QKAMP, QKAM, QKAP, and QKA treatments compared to YC. Although QKM, QK, QM, and Q treatments showed a tendency to increase vitamin C contents, there no significant differences were observed compared to CK. The addition of attapulgite and polyacrylamide might have promoted vitamin C accumulation in pakchoi. Yang et al. [74] suggested that biochar improves soil enzyme activity in the rhizosphere, as well as improving the root environment. Root growth, which plays a crucial role in nutrient uptake, significantly influences vitamin C contents in plants [75]. Besides providing trace elements, the unique crystalline structure of attapulgite can reduce soil permeability while improving water retention capacity [76,77]. In previous studies, polyacrylamide was found to improve soil water retention [78] and reduce phosphorus-leaching losses [79]. Additionally, polyacrylamide applied in combination with biochar in the soil could possibly boost the growth of plants and increase soil nutrient retention [80]. On the basis of the other growth indicators, it seems evident that biochar with attapulgite and polyacrylamide significantly improved both the nutritional quality and growth performance of pakchoi.

### 3.5. Effects of Modified Biochar on Cd Accumulation in Pakchoi

**Figure 4:**
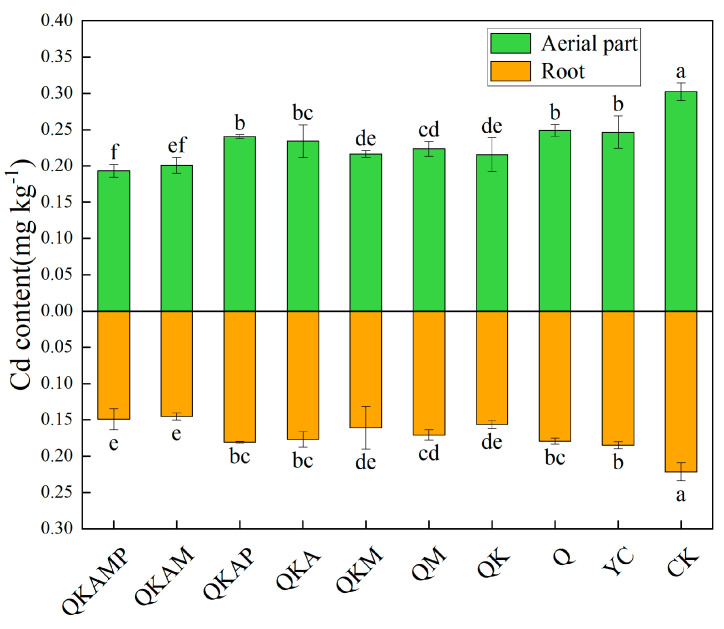
Effect of modified biochar on Cd contents in aerial parts and roots of pakchoi. Note: Error bars represent the standard deviation of triplicate samples. Different letters above or below bars, respectively, indicate a significant difference at *p* < 0.05.

As shown in Figure 4, both aerial parts and roots of pakchoi accumulate less Cd when 2% biochar is added to Cd-contaminated soil than when CK is added to the soil. Specifically, Cd contents in the aerial parts reduced by 36.1% and 33.6%, respectively, compared to CK (*p* < 0.05), and by 21.6% and 18.6%, respectively, compared to YC (*p* < 0.05) in QKAMP and QKAM treatments. Similarly, Cd contents in the roots treated with QKAMP and QKAM were reduced by 32.8% and 34.5%, respectively, compared to CK (*p* < 0.05), and by 19.5% and 21.5%, respectively, compared to YC (*p* < 0.05). Zhang et al. [81] reported that adding 5% ball-milled phosphorus-loaded biochar to Cd-contaminated soil significantly reduced the cadmium content in maize plants. According to other studies, chemical modification of biochar can enhance electrostatic interaction, thereby increasing its adsorption capacity for cadmium and reducing the transfer of cadmium to plants over bulk biochar [82]. In this study, QKAMP-modified biochar application at 2% in the Cd-contaminated alkaline soils (1.75 mg Cd kg^−1^) reduced Cd contents in both aerial and root parts of pakchoi to below 0.2 mg/kg, meeting the maximum Cd limit for leafy vegetables as set by the Chinese national Food Safety Standard for Contaminants in Food (GB 2762-2022) [83], which ensures safe vegetable production.

## 4. Conclusions

The results of this study showed that the application of modified biochar significantly increased soil pH and nutrient contents and reduced the bioavailable Cd in soils. The plant growth, nutrients content, and accumulation were also improved to varying degrees by the addition of modified biochar. Although the soluble sugar content and soluble protein contents in plants did not differ significantly from other treatments, QKAMP and QKAM significantly reduced nitrate levels and enhanced vitamin C concentrations in pakchoi compared to bulk biochar and CK. More importantly, modified biochar could reduce the accumulation of Cd in pakchoi, among which the Cd content in pakchoi for QKAMP treatment was within the safe range specified by the national standard. Based on the above results, QKAMP was recommended to be the optimal modified biochar for the safe production of pakchoi in the mild to moderate Cd-contaminated soils under weakly alkaline soil pH conditions. Further studies will explore the feasibility and stability of modified biochar across diverse soil types and varying cadmium contamination levels as well as various vegetable types in order to optimize operational and economical parameters for large-scale applications.

## Figures and Tables

**Table 1:** Tested-treatments list.

Treatment	Biochar Modification Ratio (Mass Ratio)	Treatment Abbreviation
Untreated soil	-	CK
Bulk Biochar (unmodified)	10:0	YC
Biochar (ball-milled modified)	10:0	Q
Biochar + Potassium hydroxide modified by ball milling	10:1	QK
Biochar + Calcium magnesium phosphate fertilizer modified by ball milling	10:1	QM
Biochar + Potassium hydroxide + Attapulgite modified by ball milling	10:0.5:0.5	QKA
Biochar + Potassium hydroxide + Calcium magnesium phosphate fertilizer modified by ball milling	10:0.5:0.5	QKM
Biochar + Potassium hydroxide + Attapulgite + Calcium magnesium phosphate fertilizer modified by ball milling	10:0.5:0.3:0.2	QKAM
Biochar + Potassium hydroxide +Attapulgite + Polyacrylamide (PAM1200-) modified by ball milling	10:0.5:0.5:0.005	QKAP
Biochar + Potassium hydroxide +Attapulgite + Calcium magnesium phosphate + Polyacrylamide (PAM1200-) Modified by ball milling	10:0.5:0.3:0.2:0.005	QKAMP

**Table 2:** Effect of modified biochar on the physico-chemical properties and available Cd contents in soils.

Treatments	pH	Total Nitrogen (g kg^−1^)	Organic Matter (g kg^−1^)	Available Potassium (mg kg^−1^)	Available Phosphorus (mg kg^−1^)	Available Cadmium (mg kg^−1^)
QKAMP	7.78 a	1.98 ab	77.09 b	257.13 b	42.10 c	0.83 f
QKAM	7.72 ab	1.99 a	76.00 b	258.22 b	42.63 c	0.85 ef
QKAP	7.46 d	1.99 a	78.48 ab	246.25 b	38.38 de	0.88 ef
QKA	7.49 d	1.98 a	76.47 b	253.26 b	38.99 d	0.88 de
QKM	7.71 b	1.99 a	79.86 ab	255.67 b	52.26 b	0.87 de
QM	7.63 c	1.94 b	82.65 a	90.50 c	58.18 a	0.87 de
QK	7.76 ab	1.97 ab	78.87 ab	362.50 a	35.93 ef	0.90 d
Q	7.25 e	1.97 ab	78.93 ab	83.00 c	35.82 ef	0.95 c
YC	7.28 e	1.99 a	80.21 ab	91.00 c	35.05 f	1.00 b
CK	7.07 f	1.84 c	33.58 c	74.00 c	35.41 f	1.11 a

Note: Values with the different letters in the same columns are significantly different (*p* < 0.05).

**Table 3:** Effect of modified biochar on the growth and biomass of pakchoi.

Treatments	Plant Height (cm)	Root Length (cm)	Arial Part Fresh Weight (g pot^−1^)	Root Fresh Weight (g pot^−1^)	Aerial Part Dry Weight (g pot^−1^)	Root Dry Weight (g pot^−1^)
QKAMP	21.42 ± 0.91 a	10.55 ± 0.35 a	111.85 ± 5.59 ab	12.54 ± 0.15 b	9.14 ± 0.57 a	1.97 ± 0.03 ab
QKAM	21.20 ± 0.62 ab	10.33 ± 1.06 a	109.56 ± 3.15 ab	13.78 ± 0.42 a	9.09 ± 0.45 a	2.07 ± 0.13 ab
QKAP	21.54 ± 0.61 a	10.06 ± 0.84 a	110.34 ± 8.35 ab	12.68 ± 0.30 b	8.42 ± 0.36 a	1.91 ± 0.11 abc
QKA	20.89 ± 0.18 ab	10.16 ± 1.01 a	107.13 ± 3.14 b	12.81 ± 0.33 b	8.49 ± 0.35 a	1.87 ± 0.08 bc
QKM	21.32 ± 0.26 a	9.56 ± 0.49 ab	108.73 ± 2.79 ab	13.69 ± 0.54 a	8.92 ± 0.57 a	2.11 ± 0.16 a
QM	21.35 ± 0.48 a	10.17 ± 0.58 a	116.73 ± 4.18 a	12.75 ± 0.30 b	8.80 ± 0.47 a	1.97 ± 0.13 ab
QK	21.20 ± 0.43 ab	9.75 ± 0.73 ab	108.26 ± 5.54 ab	13.23 ± 0.29 ab	8.77 ± 0.24 a	2.03 ± 0.12 ab
Q	21.35 ± 0.48 a	9.51 ± 0.67 ab	110.93 ± 3.77 ab	12.80 ± 0.40 b	8.55 ± 0.47 a	1.97 ± 0.09 ab
YC	21.28 ± 1.50 a	8.48 ± 1.00 bc	106.53 ± 4.17 b	12.73 ± 0.29 b	8.72 ± 0.43 a	1.91 ± 0.12 abc
CK	19.70 ± 0.35 b	7.96 ± 0.49 c	96.70 ± 3.79 c	11.76 ± 0.33 c	7.46 ± 0.55 b	1.71 ± 0.08 c

Note: Values with the different letters in the same columns are significantly different (*p* < 0.05).

## Data Availability

The datasets used or analyzed during the current study are available from the corresponding author upon reasonable request.
